# Hidden Blood Loss in Transforaminal Lumbar Interbody Fusion: An Analysis of Underlying Factors

**DOI:** 10.7759/cureus.35126

**Published:** 2023-02-17

**Authors:** ‏Mohammed ‏Khashab, Muath M Alswat, Adnan T Samman, Mohamed Elkhalifa

**Affiliations:** 1 Orthopedics, King Abdulaziz Medical City, Jeddah, SAU; 2 Research Office, King Abdullah International Medical Research Center, Jeddah, SAU; 3 Orthopedics, College of Medicine, King Saud bin Abdulaziz University for Health Sciences, Jeddah, SAU; 4 ‏Orthopedic Surgery, King Abdulaziz Medical City, Jeddah, SAU; 5 Orthopedic Surgery, King Abdulaziz Medical City, Jeddah, SAU

**Keywords:** blood loss, tlif, risk factors, lumbar fusion surgery, hidden blood loss

## Abstract

Background

In the management of lumbar spine diseases, various techniques have been described for minimizing intraoperative blood loss. Soft tissue extravasation and hemolysis have been referred to as hidden blood loss (HBL). By acknowledging HBL and accounting for it in our postoperative care, strategies of fluid infusion and blood transfusion may be altered. Our study aims to estimate HBL in transforaminal lumbar interbody fusion (TLIF) surgeries and to analyze associated factors.

Methods

This is a retrospective cohort study. Records of patients who underwent TLIF between January 2016 and December 2020 were reviewed. Patients with both minimally invasive (MIS) and open TLIF were included. Patients with infection, tumors, or fractures being the indication for surgery were excluded. Moreover, patients with known blood-related diseases, aged younger than 18 years, patients requiring blood transfusion, or patients with estimated intra-operative blood loss greater than 1.5 L were excluded. HBL was calculated according to the formulae depending on patients’ weight, height, and hematocrit. Statistical analyses were performed to determine associations between HBL and other factors.

Results

A total of 95 patients were included. The mean estimated blood loss (EBL) was 231 mL, whereas the mean HBL was 265 mL, and the mean total blood loss is 629.7 ml with HBL accounting for 42% of it. Significant associated factors with HBL were the type of surgery, patient’s total blood volume, preoperative hemoglobin and hematocrit, and decrease in hemoglobin and hematocrit.

Conclusion

Significant HBL may occur after TLIF, which was shown to be more than EBL. Although MIS had less EBL, it was associated with more HBL. Patients’ preoperative hemoglobin and hematocrit, and a decrease in them, have been shown to be associated with HBL. All these factors should be considered for postoperative management of blood loss.

## Introduction

In the management of degenerative lumbar spine diseases, various techniques have been described in pursuit of minimizing intra-operative blood loss and blood transfusion [1-4]. However, there remains a large number of patients who deal with anemia and its associated conditions, which did not correlate with the total blood loss measured.

Total blood loss during routine practice is measured by intra-operative bleeding and postoperative drainage, which neglects to take into account the amount of extravasation in the soft tissue and the loss from hemolysis. The concept of hidden blood loss (HBL) was first described in 2000 by Sehat et al., who reported that 26% and 49% of the total blood loss was attributed to HBL in total knee replacement and total hip replacement, respectively [5]. Smorgick et al. reported 39% of HBL from total blood loss in posterior spine fusion surgery [6]. Many studies report HBL in lumbar fusion surgeries that ranged from 227-600 mL according to Zhou et al. [7]. By retrospectively reviewing patients' medical records the aim of this study was to measure the amount of HBL in transforaminal lumbar interbody fusion (TLIF) and to analyze the associated factors.

## Materials and methods

Study design and participants

This retrospective cohort study was carried out in a tertiary hospital, King Abdulaziz Medical City, Jeddah, Saudi Arabia, and included patients who underwent TLIF that was performed between January 2016 and December 2020, and the surgery did not exceed three vertebral levels. We excluded patients with spondylodiscitis, lumber fracture, tumor, known blood-related diseases, age younger than 18 years, patients requiring blood transfusion, or patients with estimated intra-operative blood loss greater than 1.5 L. Consecutive sampling technique was used.

Surgical techniques

Open TLIF

Open TLIF was done as described by Harms and Jeszensky using a longitudinal midline incision [8]. 

Minimally Invasive Surgery (MIS)-TLIF

Above the symptomatic side, a paramidline skin incision measuring approximately 3 cm is made, then the quadrant retractor system is placed until the desired working diameter is achieved. After that, the posterolateral element of the spine is exposed. Then laminectomy and facetectomy are performed and followed by subtotal discectomy and preparation of the endplate for fusion. A cage is placed and a bone graft is packed anterior to it. Screws and rods were then placed percutaneously on both sides, and compression was applied across the cage. Thorough washing and hemostasis followed by the closure of the skin in layers are done.

Data collection 

Electronic hospital records were reviewed to collect patients’ demographic data, such as age, gender, weight, height, and comorbidities. Moreover, patients’ preoperative hemoglobin (Hgb), hematocrit (Hct), prothrombin time (PT), activated partial thromboplastin time (aPTT), and international normalized ratio (INR) were collected. Paraspinal muscle thickness and subcutaneous fat thickness were determined using pre-operative magnetic resonance imaging (MRI). Furthermore, the American Society of Anesthesiologist (ASA)'s classification and indication for surgery for each patient were also gathered. Perioperative variables such as vertebral level involved, surgical duration, estimated blood loss (EBL), and type of surgery, whether open or minimally invasive, were obtained. The immediate postoperative parameters that were collected were the length of hospital stay (LOH) and the minimum time before ambulation (TBA).

Calculating HBL

The patient’s blood volume (PBV) was calculated according to the formula of Nadler et al., which is PBV = k1 × height (m)^3^ + k2 × weight (kg) + k3 (for male: k1 = 0.3669, k2 = 0.03219, and k3 = 0.6041; for female: k1 = 0.3561, k2 = 0.03308, and k3 =0.1833) [9]. Total blood loss was then calculated by multiplying PBV by the change of Hct, according to the gross formula in which total blood loss = PBV (Hctpre−Hctpost)/Hctave, where Hctpre is the preoperative Hct, Hctpost is the postoperative Hct, nd Hctave is the average of Hctpre and Hctpost [10]. Finally, HBL was calculated according to the formula of Sehat et al., which is HBL= total blood loss − measured blood loss [5].

Statistical analyses 

Statistical analyses were performed using IBM SPSS Statistics for Windows, Version 25.0 (Released 2017; IBM Corp., Armonk, New York, United States). Data were summarized using descriptive statistics, such as mean ± standard deviation, frequency, and percentage. After examining all variables for normality by calculating the skewness and kurtosis z-values, and after using the Shapiro-Wilk test, a Pearson correlation, Student’s t-test, and ANOVA for normally distributed variables, and Spearman correlation for non-normally distributed variables, were used to determine the association between the dependent and independent variables. A p-value of less than 0.05 was considered significant.

Ethical considerations

The Institutional Review Board of King Abdullah International Medical Research Center, Jeddah, Saudi Arabia, approved the study (approval number: RJ19/090/J). Patients’ names, medical record numbers, and identifying data were not collected. Data were accessible exclusively by the authors.

## Results

A total of 95 patients (42 males and 53 females, age range 20-84 years) were included. Demographics, comorbidities, ASA classification, and subcutaneous fat and muscle thickness with the significant of their association to HBL are presented in Table 1.

**Table 1:** Patients’ details, comorbidities, and ASA classification BMI: body mass index; ASA: American Society of Anesthesiologists

Parameter	Statistics	P-value
Gender (%)		0.08
Males	44.2	
Females	55.8	
Age (years ± SD)	58.23 ± 12.5	0.34
BMI (kg/m^2^ ± SD)	31.4 ± 4.8	0.67
Subcutaneous fat thickness (mm)	31.76 ± 15.7	0.63
Muscle thickness (mm)	47.61 ± 8	0.51
Diabetes mellitus (%)	42.3	0.19
Hypertension (%)	52.6	0.45
Chronic kidney disease (%)	7.2	0.65
Coronary artery disease (%)	5.2	0.95
Smoking (%)	12.4	0.21
On antiplatelet (%)	14.4	0.79
ASA classification (%)		0.9
I	11.3	
II	72.2	
III	16.5	

Around 63% of patients had MIS-TLIF, whereas 38% had open-TLIF. Degenerative disc disease was the indication for surgery in 43.2% of patients. Moreover, the most common vertebral level involved was L4-5 (approximately 37.1% of patients). More details on indications of surgeries, levels involved in surgical details, and postoperative parameters are shown in Table 2.

**Table 2:** Surgical details and immediate postoperative parameters TLIF: transforaminal lumber interbody fusion; MIS: minimally invasive surgery

Parameter	Statistics	P-value
Time since surgery (months ± SD)	26 ± 14.6	0.23
Type of surgery		0.02
Open TLIF (%)	38.1	
MIS TLIF (%)	61.9	
Indication for surgery (%)		0.26
Degenerative disc disease	42.3	
Spinal stenosis	20.6	
Spondylolisthesis	27.8	
Prolapsed intervertebral discs	9.3	
Levels of surgery (%)		0.82
L1-L2	2.1	
L2-L3	1	
L3-L4	6.2	
L4-L5	37.1	
L5-S1	14.4	
Two levels	33	
Three levels	6.2	
Duration of surgery (hours ± SD)	3.6 ± 1.1	0.71
Drainage (ml ± SD)	497.8 ± 294.3	0.13
Hospitalization time (days ± SD)	4.49 ± 3.2	0.14
Time before ambulation (days ± SD)	1.54 ± 1.3	0.66

The mean EBL was 231.2 ± 166.4 which was less than the mean HBL (265.1 ± 411.7). Patients’ preoperative Hb and Hct were significantly associated with HBL as well as the decrease in them postoperatively. More details in blood loss and pre and postoperative laboratory investigations are shown in Table 3. 

**Table 3:** Blood loss and pre and postoperative laboratory investigations PBV: patient’s blood volume; EBL: estimated blood loss; HBL: hidden blood loss; TBL: total blood loss; Hct: hematocrit; Hb: hemoglobin; aPTT: activated partial thromboplastin time; PT: prothrombin time; INR: international normalized ratio

Parameter	Statistics	P value
PBV (L ± SD)	4.64 ± 0.77	0.03
EBL (ml ± SD)	231.2 ± 166.4	0.06
HBL (ml ± SD)	265.1 ± 411.7	
TBL (ml ± SD)	629.7 ± 411.2	0.000
Preoperative Hct ± SD	41.1 ± 4.5	0.000
Preoperative Hb (g/l ± SD)	13.3 ± 1.5	0.000
Postoperative Hct ± SD	35.8 ± 4.8	0.82
Postoperative Hb (g/l ± SD)	11.8 ± 1.6	0.99
Decrease Hct ± SD	5.3 ± 3.3	0.000
Decrease Hb (g/l ± SD)	1.5 ± 1.1	0.000
aPTT (s ± SD)	29.8 ± 4.2	0.39
PT (s ± SD)	11.9 ± 1	0.12
INR	1 ± 0.9	0.13

When comparing total blood loss between the MIS-TLIF group and the open-TLIF group, the mean total blood loss in MIS-TLIF was 471.2 ± 316 mL and in open-TLIF was 886.78 ± 421.3 mL (P˂0.00).

## Discussion

Spine surgery has seen significant technological advances over the last decades, and various methods are being used to decrease expected surgical blood loss during spinal surgery. Sehat et al. first described HBL in total hip arthroplasty; HBL accounted for 49% of the total blood loss. In their study, they highlight that HBL is thought to be due to hemolysis and extravasation into third spaces [5]. Jiang et al. found a mean of 46.8% HBL in cervical open laminoplasty, whereas Wen et al. reported a mean of 39% HBL in PSF [11,12]. Moreover, Smorgick et al. estimated HBL to be 40% [6], which was similar to our study (42% of total blood loss was attributed to HBL).

In the current study, the mean EBL was 231.2 ± 166.4 mL and the mean HBL was 265.1 ± 411.7 mL. With the HBL being more than the EBL, this shows that a significant amount of bleeding is beyond our scope of estimation, which may result in an inaccurate measurement of peri-operative blood loss. This was also noted by Ogura et al. in their study in which the HBL was 685 mL more than the mean EBL [13].

In the literature, few articles have analyzed the associated risk factors with HBL. In the present study, MIS-TLIF, preoperative low levels of hemoglobin, blood volume, and low Hct were significantly associated with an exceptional increase in HCL. With MIS-TLIF, we aimed to achieve less operative dissection, less surgical time, and better outcomes. The downside of this less invasive technique is that it limits our efforts to achieve precise homeostasis.

In a study conducted by Zhang et al., they found the MIS-TLIF technique to be associated with more HBL, which they justified by not using postoperative drainage [7]. Nevertheless, we found that MIS-TLIF showed a significantly lower total blood loss relative to open-TLIF. In our results, total blood loss in the MIS-TLIF group had approximately 50% less total blood loss than the open-TLIF group. In contrast to Lei et al., we found that a higher BMI did not significantly affect HBL, which was supported by a retrospective multicentre study by Miao et al. [14,15]. However, Goyal et al. reported that greater muscle thickness, as assessed by magnetic resonance imaging (MRI), is significantly associated with HBL [16]. Greater muscle thickness was analyzed in our study and was found to be non-significant. This could be attributed to the fact that more than half of our cohort had MIS-TLIF, which has been shown not to be associated with significant blood loss, regardless of BMI.

Our study also assessed the impact of antiplatelets on HBL. As per our preoperative preparation, all antiplatelet medications were kept on hold for seven days prior to the surgery. In this study, 15% (14 patients) were on lifelong antiplatelet medications and followed these instructions and, thus, the use of antiplatelets showed no significant association with HBL.

When we decided to go for one or two levels of TLIF, we opted for the MIS technique. In cases requiring multiple levels of TLIF, we achieved it with the open technique. The number of levels was not significant in the relation to HBL as an independent risk factor. This is due to the fact that the multiple-level surgeries were open technique, thus less HBL.

We did not find a significant association between HBL and coagulation profile because all patients had a normal coagulation profile before surgery, while those with abnormal coagulation profiles were medically optimized. Nevertheless, our study has some drawbacks. Our sample size was relatively small; larger numbers are needed for weaker correlations to be significant. Moreover, we measured Hgb and Hct on days 2 and 3 post surgery. However, as fluid shifts would not have been completed in all patients at this time, the HBL obtained might be falsely low.

## Conclusions

A significant amount of HBL may occur after TLIF, which is shown to be more than EBL. HBL was found to be correlated with patients’ preoperative Hb and Hct and was found to be significantly more elevated with MIS-TLIF. However, MIS-TLIF resulted in more HBL, and HBL was significantly lower in total blood loss relative to open-TLIF. All these factors should be considered for postoperative management of blood loss
