# Correction: Hidden Blood Loss in Transforaminal Lumbar Interbody Fusion: An Analysis of Underlying Factors

**DOI:** 10.7759/cureus.c133

**Published:** 2023-08-31

**Authors:** ‏Mohammed ‏Khashab, Muath M Alswat, Adnan T Samman, Mohamed Elkhalifa

**Affiliations:** 1 Orthopedics, King Abdulaziz Medical City, Jeddah, SAU; 2 Research Office, King Abdullah International Medical Research Center, Jeddah, SAU; 3 Orthopedics, College of Medicine, King Saud bin Abdulaziz University for Health Sciences, Jeddah, SAU; 4 ‏Orthopedic Surgery, King Abdulaziz Medical City, Jeddah, SAU; 5 Orthopedic Surgery, King Abdulaziz Medical City, Jeddah, SAU

This article has been corrected at the request of the authors to correct the affiliation for Mohammed Khashab from Orthopedic Surgery, King Abdulaziz Medical City, Jeddah, SAU to:

1. Orthopedics, King Abdulaziz Medical City, Jeddah, Saudi Arabia

2. Research Office, King Abdullah International Medical Research Center, Jeddah, Saudi Arabia.

3. Orthopedics, College of Medicine, King Saud bin Abdulaziz University for Health Sciences, Jeddah, Saudi Arabia

The authors deeply regret that these errors were not identified and addressed prior to publication.

 

